# Gallic Acid Attenuates Ifosfamide-Induced Gut Microbiota Dysbiosis: A Full-Length 16S rRNA Amplicon Sequencing Study

**DOI:** 10.3390/microorganisms14071537

**Published:** 2026-07-14

**Authors:** Cihat Öztürk, Süleyman Yalçın, Sadık Küçükgünay, Hakan Farzin Mehmetzade, Memiş Bolacalı, Elif Sevim

**Affiliations:** 1 Department of Medical Microbiology, Faculty of Medicine, Kırşehir Ahi Evran University, 40100 Kırşehir, Türkiye; 2 Turkish Public Health Directorate General Molecular Microbiology Reference Laboratory, 06080 Ankara, Türkiye; slymn_yalcin@hotmail.com (S.Y.); hfmz1986@gmail.com (H.F.M.); 3 Department of Medical Pharmacology, Faculty of Medicine, Kırşehir Ahi Evran University, 40100 Kırşehir, Türkiye; sadik.kucukgunay@ahievran.edu.tr; 4 Department of Biostatistics and Medical Informatics, Faculty of Medicine, Kırşehir Ahi Evran University, 40100 Kırşehir, Türkiye; memis.bolacali@ahievran.edu.tr; 5 Department of Medical Biology, Faculty of Medicine, Kırşehir Ahi Evran University, 40100 Kırşehir, Türkiye; esevim@ahievran.edu.tr

**Keywords:** ifosfamide, gallic acid, dysbiosis, 16S rRNA amplicon sequencing

## Abstract

Chemotherapy-induced gastrointestinal toxicity represents a major clinical challenge, with accumulating evidence implicating gut microbiota dysbiosis in reduced treatment tolerance. Ifosfamide is a widely used alkylating agent; however, its effects on gut microbial community structure remain incompletely understood. This preclinical study investigated ifosfamide-associated microbiota alterations and evaluated the microbiota-modulating potential of gallic acid as a supportive intervention. Male rats were assigned to control, ifosfamide, gallic acid, and combined ifosfamide + gallic acid groups. Fecal samples were collected longitudinally and analyzed using full-length 16S rRNA gene sequencing for high-resolution taxonomic profiling. At the phylum level, ifosfamide exposure induced a marked decrease in Bacillota accompanied by a significant expansion of Bacteroidota, reflecting a dysbiotic shift associated with intestinal stress. Genus-level analysis revealed substantial reductions in beneficial taxa, including *Lactobacillus*, *Ligilactobacillus*, and *Blautia*, alongside enrichment of stress-adaptive and opportunistic genera such as *Romboutsia* and *Segatella*. Species-level profiling demonstrated significant depletion of mucosa-associated lactic acid bacteria, including *Lactobacillus johnsonii*, *Lactobacillus intestinalis*, and *Ligilactobacillus murinus*, following ifosfamide treatment. Conversely, opportunistic taxa such as *Romboutsia ilealis* and *Segatella copri* and transient increases in *Escherichia coli* were observed, consistent with chemotherapy-induced intestinal perturbation. Gallic acid administration partially preserved microbial diversity, attenuated the expansion of opportunistic taxa, and supported recovery of beneficial bacteria, resulting in an intermediate microbial profile under combination treatment. These findings provide preclinical evidence that gallic acid mitigates ifosfamide-associated gut dysbiosis and highlight gut microbiota modulation as a potential adjunctive strategy to improve chemotherapy tolerance. Further translational and clinical investigations are warranted.

## 1. Introduction

Chemotherapy remains a cornerstone in cancer management, employing diverse mechanisms such as DNA alkylation, antimetabolite incorporation, and topoisomerase inhibition. Among these, alkylating agents, including cyclophosphamide and ifosfamide (IFO), represent some of the most effective cytotoxic drugs used against sarcomas, lymphomas, and germ cell tumors [1]. However, their off-target toxicity, including nephrotoxicity, neurotoxicity, and mucositis, limits therapeutic benefit due to nonspecific interactions with healthy tissues [2].

The gastrointestinal microbiome, comprising over 10^14^ microorganisms, functions as a metabolic and immunomodulatory organ that influences drug metabolism, immune signaling, and inflammation control [3]. Microbes can bioactivate or inactivate anticancer agents, modulate pharmacokinetics, and determine systemic responses to therapy [4].

Chemotherapeutic regimens profoundly disrupt gut microbial homeostasis, often depleting beneficial taxa such as *Lactobacillus*, *Bifidobacterium*, and *Akkermansia* while promoting opportunistic pathogens including *Enterococcus* and *Clostridium* [2,5]. In particular, IFO has been associated with intestinal and neurological toxicity, partly mediated by microbial dysbiosis and accumulation of toxic metabolites such as chloroacetaldehyde [1]. IFO-induced dysbiosis disrupts the Firmicutes/Bacteroidota ratio, depleting beneficial genera such as *Lactobacillus*, *Bifidobacterium*, and *Akkermansia* while promoting opportunistic pathogens including *Enterococcus* and *Clostridium*. These microbial shifts contribute to oxidative stress, mucosal injury, and immune imbalance through pathways like TLR4/NF-κB activation [6]. Restoration of *Akkermansia* and *Lactobacillus* populations has been shown to alleviate inflammation and reinforce intestinal barrier integrity, highlighting the therapeutic potential of microbiota-targeted interventions against IFO-induced toxicity [7].

In this regard, natural polyphenolic compounds, notably gallic acid (GA), have drawn attention for their ability to modulate the gut microbiota and attenuate inflammation [8]. Gallic acid, found in grapes, tea, and pomegranates, exhibits antioxidant and microbiota-modulating properties that may counteract chemotherapy-induced intestinal damage [9].

The integration of microbiota-targeted adjuncts into oncology represents an emerging precision-medicine approach. Maintaining microbial diversity during chemotherapy may not only alleviate gastrointestinal toxicity but also enhance therapeutic efficacy via improved immune and metabolic resilience [10].

Therefore, the present study aims to investigate the impact of IFO on gut microbial diversity and composition in a rat anticancer model, and to evaluate whether gallic acid co-administration can alleviate IFO-induced dysbiosis. Using full-length 16S rRNA amplicon sequencing, this study seeks to elucidate the microbial alterations associated with chemotherapy and assess the potential of gallic acid to restore intestinal homeostasis and reduce treatment-related toxicity.

## 2. Materials and Methods

### 2.1. Animal Experimental Model

All experimental protocols were approved by the Kırşehir Ahi Evran University Local Animal Experiments Ethics Committee (Approval No: 2026/10-10) and were conducted in accordance with the Animal Research: Reporting of In Vivo Experiments guidelines and international standards for animal welfare. A total of twenty male Albino Wistar rats (14 weeks old, 270–320 g) were obtained from the Kırşehir Ahi Evran University Experimental Animal Research Laboratory (Kırşehir, Türkiye). Clinical trial number: not applicable. Animals were housed under controlled environmental conditions (temperature: 22 ± 2 °C, relative humidity: 55 ± 10%, 12 h light/dark cycle) with ad libitum access to standard pellet diet and tap water.

During the experimental period, animals were observed daily for behavioral changes, signs of toxicity, diarrhea, and mortality. On day 6, all rats were anesthetized using xylazine (10 mg/kg, i.p.) and ketamine (60 mg/kg, i.p.) and euthanized under deep anesthesia in accordance with institutional animal welfare regulations (Table 1).

### 2.2. Sample Collection and Full-Length 16S rRNA Amplicon Sequencing

Fresh fecal samples were aseptically collected from each rat on day 3 and day 6. Samples were immediately stored at −80 °C until microbial DNA extraction.

Microbial DNA was extracted from approximately 200 mg of fecal material using the QIAamp Fast DNA Stool Mini Kit (Qiagen GmbH, Hilden, Germany) according to the manufacturer’s instructions, with optimized lysis steps to ensure efficient microbial cell disruption [13]. DNA concentration and purity were assessed using a Qubit (Thermo Fisher Scientific, Waltham, MA, USA).

The full-length 16S rRNA gene was amplified using universal primers (Table 2) and Phanta Max Super-Fidelity DNA Polymerase (Vazyme, Nanjing, China). PCR conditions were: 95 °C for 3 min, followed by 30 cycles of 95 °C for 15 s, 58 °C for 15 s, and 72 °C for 45 s, with a final extension at 72 °C for 5 min. PCR products were evaluated by 1% agarose gel electrophoresis.

Library preparation was performed using the Ligation Sequencing Kit (SQK-NBD114.24) from Oxford Nanopore Technologies (Oxford Nanopore Technologies, Oxford, UK) following the manufacturer’s protocol for barcode ligation, adapter addition, and bead-based clean-up steps. Sequencing was conducted on a GridION platform (Oxford Nanopore Technologies, Oxford, UK) with R10.4 flow cells. Raw reads were base-called using Dorado v0.7.0 (Oxford Nanopore Technologies, Oxford, UK), demultiplexed by barcodes, and quality-filtered. Sequences between 1250 and 1750 bp were retained for downstream analysis. Taxonomic assignment was carried out using Kraken2 v2.1.3 with the SILVA 138 reference database for bacterial classification [14]. Microbial diversity analyses were performed using Python v3.10-based statistical libraries including Pandas v2.0, NumPy v1.24, SciPy v1.11, and statsmodels v0.14 based on Quast et al. [15].

### 2.3. Statistical Analysis

The research data were analysed using IBM SPSS Statistics Standard Concurrent User V 29 (IBM Corp., Armonk, NY, USA). Microbiota characteristics were expressed as mean, standard deviation, minimum and maximum values for continuous variables. The normality of the variables was assessed by examining skewness and kurtosis values, histograms, and Q-Q plots; the data were found to be normally distributed. To examine group differences in microbiota composition across the Con, IFO, GA, and IFO + GA groups over time, mixed-design ANOVA was applied. A *p*-value of less than 0.05 was considered statistically significant. Beta diversity was assessed using Permutational Multivariate Analysis of Variance (PERMANOVA) with Bray–Curtis dissimilarity.

## 3. Results

### 3.1. Alpha Diversity

**Figure 1:**
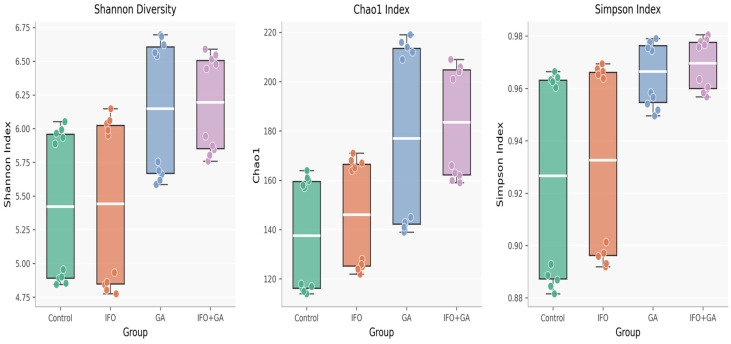
Comparative alpha diversity indices across experimental groups and time points. Shannon, Chao1, and Simpson indices are presented for each group (Con, IFO, GA, IFO + GA) at Day 3 (Time A) and Day 6 (Time B). Statistical comparisons were performed using mixed-design ANOVA with Bonferroni post-hoc correction. Bars represent mean values; error bars indicate standard deviation.

Alpha diversity indices revealed distinct alterations in microbial richness and evenness among experimental groups (Figure 1).

At T1 (Day 3), Shannon diversity rankings from highest to lowest were IFO + GA > GA > Control > IFO (*p* < 0.001), indicating that IFO administration alone was associated with the lowest microbial diversity at the initial sampling point. Chao1 richness followed a similar pattern, with IFO + GA > GA > IFO > Control (*p* < 0.001), while Simpson evenness rankings were likewise IFO + GA > GA > IFO > Control (*p* < 0.001). Notably, the IFO group ranked lowest among all groups across Shannon, Chao1, and Simpson indices at T1 (Table S1).

At T2 (Day 6), diversity rankings shifted substantially across all indices. Shannon diversity ranked GA > IFO > Control > IFO + GA (*p* < 0.001); Chao1 ranked GA > IFO > IFO + GA > Control (*p* < 0.001); and Simpson ranked GA > IFO > Control > IFO + GA (*p* < 0.001). Diversity rankings differed between T1 and T2 across all treatment groups (Table S2).

The group × time interaction was statistically significant and of large magnitude for all indices (Shannon: *p* < 0.001; Chao1: *p* < 0.001; Simpson: *p* < 0.001), confirming that diversity trajectories differed significantly among treatment groups. Notably, GA administration was associated with consistently high diversity values across both sampling points, whereas the IFO + GA group exhibited the highest diversity at T1 followed by a marked decline at T2 across all indices (Table S3).

### 3.2. Principal Coordinate Analysis

**Figure 2:**
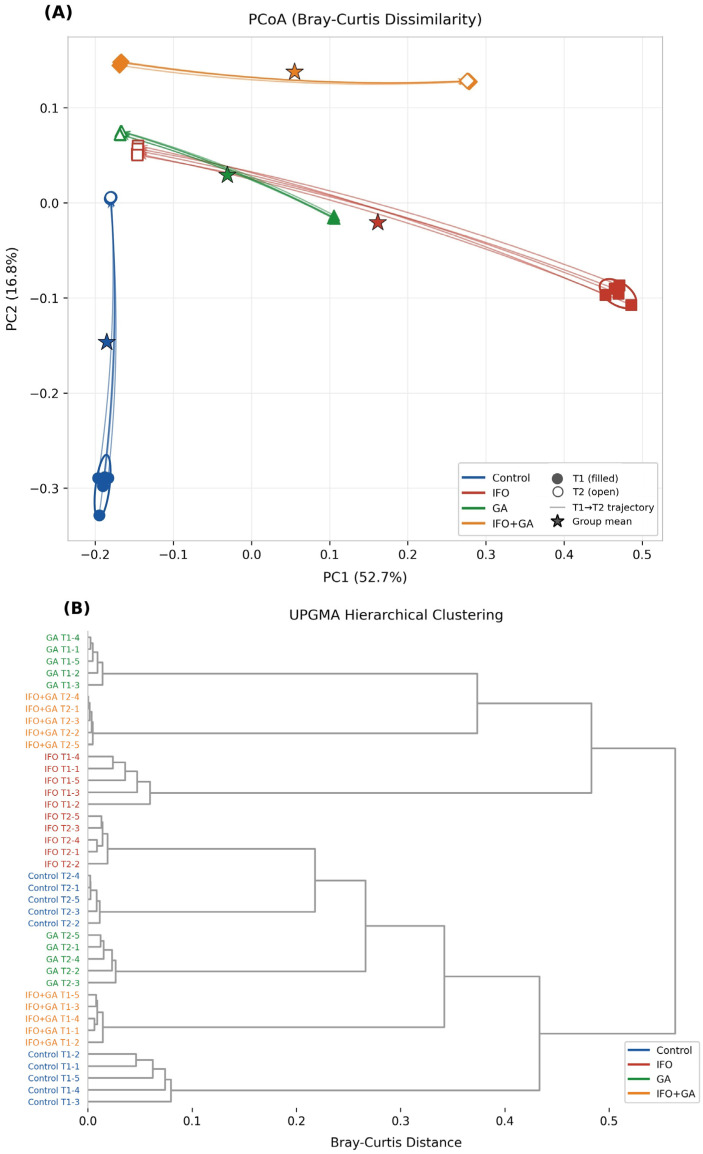
Beta diversity comparisons of gut microbiomes. (**A**) Principal Coordinate Analysis (PCoA) based on Bray–Curtis dissimilarity. Each point represents an individual animal sample. Filled symbols indicate T1 (Day 3) and open symbols indicate T2 (Day 6). Stars (★) indicate group centroids. Solid and dashed ellipses represent 95% confidence intervals for T1 and T2, respectively. Arrows connect individual animals from T1 to T2 (longitudinal trajectories). PERMANOVA: T1: *p* < 0.001; T2: *p* < 0.001. (**B**) UPGMA hierarchical clustering dendrogram based on Bray–Curtis distance matrix. Each leaf represents an individual animal; leaf labels are colored by treatment group. PC1, first principal coordinate; PC2, second principal coordinate; UPGMA, unweighted pair group method with arithmetic mean.

PCoA based on Bray–Curtis dissimilarity revealed distinct clustering among treatment groups (Figure 2). The IFO group clustered separately from the Con and GA groups along the first principal coordinate (PC1), whereas partial overlap was observed between the GA and IFO + GA groups.

### 3.3. Taxonomic Composition at the Phylum, Genus and Species Levels

**Figure 3:**
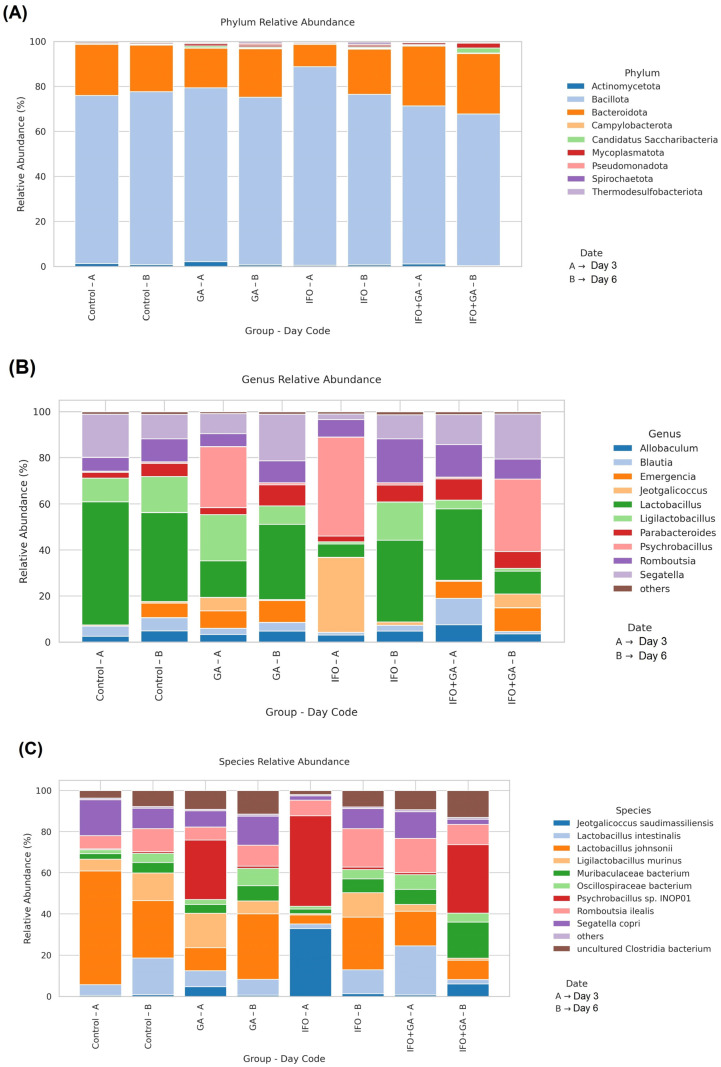
Relative abundance of gut microbiota at phylum (**A**), genus (**B**), and species (**C**) levels across treatment groups and time points. Each bar represents the mean relative abundance (%) of the dominant taxa for each group. T1: Day 3; T2: Day 6. Groups: Control, GA (gallic acid), IFO (ifosfamide), IFO + GA (ifosfamide + gallic acid). Taxa with relative abundance <1% across all groups are grouped as ‘others’.

Taxonomic composition was analyzed at the phylum, genus, and species levels (Figure 3). At the phylum level, IFO treatment induced a statistically significant decline in Bacillota, accompanied by a reciprocal expansion of Bacteroidota (group effect: *p* < 0.001; group × time interaction: *p* < 0.001). Bacteroidota abundance increased in the IFO group from Day 3 to Day 6 (group effect: *p* < 0.001; group × time interaction: *p* < 0.001). In the IFO + GA group, the decline in Bacillota was less pronounced compared to IFO alone, and the expansion of Bacteroidota was partially restrained (Table S4).

At the genus level, IFO treatment was associated with reduced abundance of *Lactobacillus*, *Ligilactobacillus*, and *Blautia*. *Lactobacillus* showed the strongest group effect of all genera analyzed (*p* < 0.001), with the lowest abundance observed in the IFO group at Day 3, followed by a partial recovery at Day 6. *Ligilactobacillus* was most depleted in the IFO group at Day 3 (group effect: *p* < 0.001). *Blautia* showed the lowest abundance in the IFO group, with a strong group effect (*p* < 0.001). GA administration alone was associated with higher abundance of all three genera, while the IFO + GA group showed intermediate abundance levels (Table S5).

Conversely, *Romboutsia*, *Segatella*, *Enterococcus*, and *Escherichia* showed increased abundance under IFO exposure. *Romboutsia* abundance was markedly elevated in the IFO group at both time points (*p* < 0.001). *Segatella* abundance increased significantly in the IFO group at Day 6 (*p* < 0.001). *Enterococcus* showed the most pronounced temporal increase under IFO treatment (*p* < 0.001), rising substantially from Day 3 to Day 6. *Escherichia* abundance also increased in the IFO group (*p* < 0.001). GA co-administration was associated with attenuated expansion of *Romboutsia*, *Enterococcus*, and *Escherichia*, with the IFO + GA group maintaining intermediate abundance levels relative to IFO and GA groups.

High-resolution species-level analysis confirmed and extended the genus-level findings (Figure 3C). Among beneficial lactic acid bacteria, *Lactobacillus johnsonii* (*L. johnsonii*) and *Ligilactobacillus murinus*—species with established mucosal-protective and anti-inflammatory roles—were most depleted in the IFO group at Day 3 (*L. johnsonii*: p < 0.001; *L. murinus*: p < 0.001), while GA treatment alone preserved or enhanced their abundance. *Lactobacillus intestinalis* (*L. intestinalis*) showed a comparable pattern (*p* < 0.001), with lowest abundance in the IFO group and highest in the GA and IFO + GA groups at Day 3, followed by a temporal shift at Day 6 reflecting community-level reorganization. *Romboutsia ilealis* was markedly elevated in both IFO and IFO + GA groups at Day 3 (*p* < 0.001), consistent with its genomic specialization for fermentative and redox-adaptive metabolism under mucosal stress. *Segatella copri* increased substantially in the IFO group at Day 6 (*p* < 0.001), consistent with its known capacity to proliferate under inflammatory mucosal conditions. *Escherichia coli* showed a transient increase under IFO treatment at Day 3, which was attenuated by GA co-administration (*p* < 0.001), reflecting the antimicrobial and microbiota-stabilizing properties of gallic acid against facultative anaerobes. *Enterococcus hirae*, while showing a modest group effect compared to genus-level *Enterococcus* data (*p* = 0.008), demonstrated a consistent temporal increase across IFO-treated groups, further supporting the pattern of opportunistic expansion under chemotherapeutic stress (Table S6).

## 4. Discussion

The present study demonstrated that ifosfamide administration induced significant alterations in gut microbial diversity and composition, while gallic acid co-administration modified several of these changes. These findings are consistent with accumulating evidence indicating that chemotherapeutic agents can substantially reshape intestinal microbial communities and contribute to treatment-associated dysbiosis [2,4,16]. Significant changes were observed in both alpha- and beta-diversity metrics, indicating that IFO exposure affected not only individual taxa but also the overall structure of the microbial community.

Chemotherapy-associated alterations in gut microbial diversity have been consistently reported across different patient populations and experimental models. Rattanathammethee et al. [17] demonstrated significant reductions in Shannon and Simpson diversity indices during the febrile neutropenic period in AML patients receiving intensive chemotherapy. Similarly, Han et al. [18] reported a progressive decline in gut microbial alpha diversity in colon cancer patients undergoing 5-fluorouracil-based chemotherapy, accompanied by shifts in dominant bacterial phyla. In an experimental rat model, Wu et al. [19] also observed marked reductions in Chao1 and Shannon diversity indices following cisplatin administration, with partial attenuation after co-administration of a protective agent. Consistent with these observations, IFO administration in the present study was associated with the lowest alpha diversity values at the initial sampling point and significant temporal changes across all diversity indices.

Beyond alpha diversity, the observed differences in overall microbial community composition indicate that IFO exposure was associated with broad alterations in the taxonomic structure of the gut microbiota. Similar community-level shifts have been reported following exposure to various chemotherapeutic agents in both experimental and clinical settings [4,16], supporting the concept that chemotherapy can influence microbial community organization beyond changes in individual taxa. Notably, GA administration was associated with consistently higher diversity values throughout the study period, in agreement with previous reports describing polyphenols as modulators of gut microbial composition through interactions with microbial metabolism and community ecology [8,20]. However, the functional consequences of these compositional alterations cannot be determined from the present taxonomic data alone and require further investigation.

At the phylum level, IFO treatment was associated with a decline in Bacillota and a reciprocal expansion of Bacteroidota, a pattern previously reported in chemotherapy-associated intestinal dysbiosis [2,4,16]. Although the biological significance of alterations in the Bacillota/Bacteroidota balance remains context-dependent, these shifts suggest substantial restructuring of dominant microbial community members. GA co-administration modified these phylum-level responses, with the IFO + GA group exhibiting a less pronounced decline in Bacillota abundance compared with IFO treatment alone.

At the genus level, IFO treatment was associated with depletion of *Lactobacillus*, *Ligilactobacillus*, and *Blautia*, genera commonly associated with stable and health-associated gut microbial communities in previous studies [21,22]. *Lactobacillus* showed one of the strongest treatment-related responses, with the lowest abundance observed in the IFO group at Day 3 followed by partial recovery at Day 6, a pattern consistent with cisplatin-induced *Lactobacillus* depletion and subsequent partial recovery reported by Wu et al. [19] in a rat model. Similar variable responses of *Lactobacillus* populations to chemotherapeutic exposure have previously been reported and may depend on the specific drug, host environment, and baseline microbial composition [23]. *Blautia* was likewise reduced following IFO treatment, consistent with reports linking this genus to stable intestinal microbial communities [22].

Conversely, opportunistic and stress-associated genera including *Enterococcus*, *Romboutsia*, and *Segatella* expanded under IFO exposure. *Enterococcus* exhibited a pronounced temporal increase, consistent with previous observations of *Enterococcus* enrichment during chemotherapy-associated dysbiosis in both experimental models and clinical settings and with the known ecological resilience of enterococci under environmental stress conditions [16,17]. Similar increases in *Romboutsia* and *Segatella* have been described under conditions of intestinal ecological disturbance and may reflect adaptation to altered nutrient availability and gut environmental conditions [24,25,26]. Nevertheless, causal relationships cannot be established from taxonomic data alone.

GA administration, either alone or in combination with IFO, was associated with higher abundances of *Lactobacillus*, *Ligilactobacillus*, and *Blautia*, together with reduced expansion of *Enterococcus* and *Romboutsia* relative to IFO treatment alone. These findings are consistent with previous reports describing polyphenolic compounds, including gallic acid, as modulators of gut microbial ecology [8,9,20]. Although the precise mechanisms underlying these observations remain unclear, GA may influence microbial community composition through selective effects on bacterial growth and ecological interactions within the gut microbiota. The functional implications of these GA-associated microbiota changes, however, remain to be determined and should be addressed in future studies.

At the species level, *L. johnsonii*, *L. murinus*, and *L. intestinalis* were among the most depleted taxa in the IFO group, whereas *Romboutsia ilealis* and *Segatella copri* showed marked expansion. These findings extend the genus-level observations and provide higher-resolution insight into taxa most affected by IFO-associated perturbation and most responsive to GA co-administration. The use of full-length 16S rRNA sequencing enabled direct species-level taxonomic assignment and represents an important methodological advantage over conventional short-read approaches [27,28,29].

The growing interest in pharmacomicrobiomics has emphasized the importance of understanding interactions between anticancer agents and the gut microbiota [3,4]. The present findings contribute to this field by providing taxonomic evidence of IFO-associated dysbiosis and its modulation by GA. However, the functional implications of the observed microbial alterations remain to be determined and should be addressed in future studies integrating microbiome composition with host physiological and metabolic outcomes.

Although the present study provides novel insights into IFO-associated microbiota alterations and their modulation by GA, several limitations should be acknowledged. The relatively small sample size, while consistent with institutional ethical guidelines and the 3R principles and comparable to similar exploratory rodent microbiome studies in the literature, limits the statistical power and generalizability of the present findings. Additionally, the use of healthy rather than tumor-bearing animals, while enabling controlled assessment of chemotherapy-associated microbiota changes in isolation from tumor-related confounding factors, may not fully reflect the clinical setting in which IFO is administered to oncology patients. Furthermore, as the present study focused on taxonomic characterization, the functional consequences of the observed microbial alterations, including potential changes in metabolite production, intestinal barrier function, and host immune responses, were not directly assessed and remain to be elucidated in future investigations. Studies employing larger cohorts, tumor-bearing models, and complementary functional approaches including metagenomics and metabolomics will be necessary to fully characterize the biological significance of the microbiota changes identified herein.

## 5. Conclusions

In conclusion, ifosfamide administration was associated with significant, time-dependent alterations in gut microbial diversity and composition, indicating substantial restructuring of the intestinal microbial ecosystem. Co-administration of gallic acid modified several of these microbial changes across multiple taxonomic levels, suggesting that dietary polyphenols may influence microbiota responses during chemotherapy. Collectively, these findings contribute to the growing understanding of chemotherapy-associated dysbiosis and provide new insights into the interaction between alkylating chemotherapeutic agents and the gut microbiota, with particular emphasis on the potential of gallic acid as a microbiota-modulating dietary compound during chemotherapy. Furthermore, these findings underscore the importance of future large-scale studies integrating shotgun metagenomic sequencing and complementary multi-omics approaches to achieve a more comprehensive understanding of the complex interactions among chemotherapy, the gut microbiota, and host responses.

## Figures and Tables

**Table 1:** Animal experimental design and treatment protocol.

Experimental Groups	Treatment Protocol
Control (Con)	No intervention was applied to this group.
IFO	Received IFO (Holoxan 500 mg, Baxter Oncology GmbH, Halle, Germany) at a dose of 50 mg/kg/day via intraperitoneal (i.p.) injection for five consecutive days.
Gallic Acid (GA)	Received GA (3,4,5-trihydroxybenzoic acid; purity ≥98%; Sigma-Aldrich, St. Louis, MO, USA) orally at a dose of 50 mg/kg/day for five consecutive days.
IFO + GA	Administered 50 mg/kg/day GA orally, followed 0.5–1 h later by 50 mg/kg/day IFO via i.p. injection for five consecutive days [11,12].

**Table 2:** Universal bacterial 16S rRNA primers used for full-length amplification.

Primer	Sequence (5′-3′)	
27F	AGAGTTTGATYMTGGCTCAG	Binds at positions 8–27; targets most bacterial species.
1492R	GGTTACCTTGTTACGACTT	Binds at positions 1510–1492; universal for bacterial amplification.

## Data Availability

The original contributions presented in this study are included in the article/Supplementary Materials. Further inquiries can be directed to the corresponding author.

## Supplementary Materials

The following supporting information can be downloaded at: https://www.mdpi.com/article/10.3390/microorganisms14071537/s1, Table S1: Effect of group × time interaction on Shannon diversity index; Table S2: Effect of group × time interaction on Chao1 richness index; Table S3: Effect of group × time interaction on Simpson evenness index; Table S4: Phylum-level statistical analysis of gut microbiota composition across experimental groups and time points (T1 and T2); Table S5: Genus-level statistical analysis of gut microbiota composition across experimental groups and time points (T1 and T2); Table S6: Species-level statistical analysis of gut microbiota composition across experimental groups and time points (T1 and T2).
