# Supplementary material for: Gallic Acid Attenuates Ifosfamide-Induced Gut Microbiota Dysbiosis: A Full-Length 16S rRNA Amplicon Sequencing Study
Source: Microorganisms. 2026 Jul 14;14(7):1537. doi: 10.3390/microorganisms14071537 (PMC13413710; doi:10.3390/microorganisms14071537)
Supplement: Supplementary file 1 [file microorganisms-14-01537-s001.zip › microorganisms-4369278-supplementary.pdf]

Supplementary Tables

Table S1, Effect of group × time interaction on Shannon diversity index,			
**Group**	**T1 (Day 3) Mean ± SD**	**T2 (Day 6) Mean ± SD**	**Test Statistics**
**Control**	$4,889 \pm 0,044^{c}$	$5,9668 \pm 0,0625^{bc}$	t = 91,899; p < 0,018; $p\eta^2 = 0,026$
**IFO**	$4,8429 \pm 0,0598^{c}$	$6,0371 \pm 0,0751^{b}$	t = 174,949; p < 0,001; $p\eta^2 = 0,015$
**GA**	$5,6614 \pm 0,0652^{b}$	$6,6215 \pm 0,0693^{a}$	t = 99,492; p < 0,013; $p\eta^2 = 0,022$
**IFO+GA**	$6,5148 \pm 0,0568^{a}$	$5,8446 \pm 0,0706^{c}$	t = 99,977; p < 0,001; $p\eta^2 = 0,015$
**Test Statistics**	F = 957,12; p < 0,001; $p\eta^2 = 0,994$	F = 123,34; p < 0,001; $p\eta^2 = 0,959$	
Group Effect: F = 224,12; p < 0,001; $p\eta^2 = 0,977$ | Time Effect: F = 20193,18; p < 0,001; $p\eta^2 = 0,999$ | Group × Time Effect: F = 9506,60; p < 0,001; $p\eta^2 = 0,999$			

t: paired t-test; F: Mixed Design ANOVA; $p\eta^2$: partial eta squared, Superscripts (a > b > c > d) indicate statistically significant differences between group means at the same time point (p < 0,05), Data are presented as mean ± standard deviation (SD), IFO: ifosfamide; GA: gallic acid; IFO+GA: ifosfamide + gallic acid,

**Table S2, Effect of group × time interaction on Chao1 richness index,**			
**Group**	**T1 (Day 3) Mean ± SD**	**T2 (Day 6) Mean ± SD**	**Test Statistics**
**Control**	116,00 ± 1,58[d]	160,00 ± 2,74[c]	t = 80,333; p < 0,003; pη² = 1,225
**IFO**	125,00 ± 2,24[c]	167,00 ± 2,74[b]	t = 132,816; p < 0,003; pη² = 0,707
**GA**	142,00 ± 2,24[b]	214,00 ± 3,81[a]	t = 101,823; p < 0,001; pη² = 1,581
**IFO+GA**	205,00 ± 2,92[a]	162,00 ± 2,74[bc]	t = 135,978; p < 0,006; pη² = 0,707
**Test Statistics**	F = 1354,17; p < 0,001; pη² = 0,997	F = 356,17; p < 0,001; pη² = 0,985	
Group Effect: F = 372,06; p < 0,001; pη² = 0,986 | Time Effect: F = 13225,00; p < 0,001; pη² = 0,999 | Group × Time Effect: F = 61888,96; p < 0,001; pη² = 0,999			

t: paired t-test; F: Mixed Design ANOVA; pη²: partial eta squared, Superscripts (a > b > c > d) indicate statistically significant differences between group means at the same time point (p < 0,05), Data are presented as mean ± standard deviation (SD), IFO: ifosfamide; GA: gallic acid; IFO+GA: ifosfamide + gallic acid,

Table S3, Effect of group × time interaction on Simpson evenness index,			
**Group**	**T1 (Day 3) Mean ± SD**	**T2 (Day 6) Mean ± SD**	**Test Statistics**
**Control**	0,8869 ± 0,0043[d]	0,9634 ± 0,0022[bc]	t = 81,411; p < 0,001; pη² = 0,002
**IFO**	0,8959 ± 0,0037[c]	0,9665 ± 0,0021[b]	t = 96,342; p < 0,002; pη² = 0,002
**GA**	0,9541 ± 0,0035[b]	0,9767 ± 0,0018[a]	t = 28,748; p < 0,001; pη² = 0,002
**IFO+GA**	0,9779 ± 0,0018[a]	0,9598 ± 0,0025[c]	t = 55,594; p < 0,001; pη² = 0,001
**Test Statistics**	F = 821,75; p < 0,001; pη² = 0,994	F = 55,59; p < 0,001; pη² = 0,912	
Group Effect: F = 334,58; p < 0,001; pη² = 0,984 | Time Effect: F = 10719,48; p < 0,001; pη² = 0,999 | Group × Time Effect: F = 3687,40; p < 0,001; pη² = 0,999			

t: paired t-test; F: Mixed Design ANOVA; pη²: partial eta squared, Superscripts (a > b > c > d) indicate statistically significant differences between group means at the same time point (p < 0,05), Data are presented as mean ± standard deviation (SD), IFO: ifosfamide; GA: gallic acid; IFO+GA: ifosfamide + gallic acid,

Table S4. Phylum-level statistical analysis of gut microbiota composition across experimental groups and time points (T1 and T2).

	Bacillota_T1_17.10.2024	Bacillota_T2_21.10.2024	Test Statistics
Control	74.64±0.41[b] (74.12 - 75.14)	76.93±0.08[a] (76.8 - 77.02)	t=12.512 p<0.001 pη2=5.108
İfosfamide	88.15±0.25[a] (87.88 - 88.49)	70.85±3.43[c] (66.01 - 75.83)	t=12.212 p<0.001 pη2=4.986
Gallic Asid	73.51±3.46[b] (68.92 - 77.22)	74.29±1.39[b] (72.15 - 76.11)	t=0.452 p=0.670 pη2=0.184
İfosfamide+ Gallic Asid	70.14±0.89[c] (68.91 - 71.35)	67.38±1.41[d] (65.12 - 69.04)	t=5.337 p=0.003 pη2=2.179
Test Statistics	F=116.234 p<0.001 pη2=0.976	F=26.427 p<0.001 pη2=0.799	
Statistical Model	Group Effect: F=75.818 p<0.001 pη2=0.919<br>Time Effect: F=54.237 p<0.001 pη2=0.731<br>Grup*Time Effect: F=60.249 p<0.001 pη2=0.900		
	Bacteroidata_T1_17.10.2024	Bacteroidata_T2_21.10.2024	
Control	22.79±0.17[b] (22.61 - 23.02)	20.69±0.09[bc] (20.59 - 20.83)	t=24.410 p<0.001 pη2=9.966
İfosfamide	9.82±0.41[d] (9.12 - 10.28)	20.09±1.03[c] (18.21 - 21.01)	t=38.724 p<0.001 pη=15.813
Gallic Asid	16.10±1.28[c] (14.31 - 17.64)	21.56±0.73[b] (20.41 - 22.38)	t=8.131 p<0.001 pη2=3.319
İfosfamide+ Gallic Asid	25.41±2.45a (20.54 - 27.18)	26.76±1.12[a] (25.11 - 28.34)	t=1.211 p=0.280 pη2=0.494
Test Statistics	F=150.549 p<0.001 pη2=0.958	F=77.772 p<0.001 pη2=0.921	
Statistical Model	Group Effect: F=196.068 p<0.001 pη2=0.967<br>Time Effect: F=126.929 p<0.001 pη2=0.864<br>Grup*Time Effect: F=64.543 p<0.001 pη2=0.906		
	Diğer_T1_17.10.2024	Diğer_T2_21.10.2024	
Control	2.57±0.24[b] (2.25 - 2.86)	2.39±0.01[c] (2.37 - 2.40)	t=1.824 p=0.128 pη2=0.745
İfosfamide	2.03±0.62[b] (1.37 - 2.97)	9.07±3.26[a] (4.06 - 13.10)	t=8.309 p=0.003 pη2=2168
Gallic Asid	10.39±4.62[a] (5.14 - 15.97)	4.15±2.07[bc] (1.51 - 7.44)	t=2.651 p=0.045 pη2=1.082
İfosfamide+ Gallic Asid	4.45±2.73[b] (1.47 - 9.23)	5.87±2.35[b] (3.75 - 9.77)	t=1.046 p=0.343 pη2=0.427
Test Statistics	F=12.033 p<0.001 pη2=0.643	F=9.548 p<0.001 pη2=0.589	
Statistical Model	Group Effect: F=8.502 p<0.001 pη2=0.560<br>Time Effect: F=0.453 p=0.508 pη2=0.022<br>Grup*Time Effect: F=13.046 p<0.001 pη2=0.662		

t: paired simple t-test, KW: Kruskal-Wallis, Z: Wilcoxon, F: Mixed Design ANOVA, pη2: Partial Eta Squared, a>b>c>d: Differences between means with different letters in the same row or column are significant (p<0.05). Descriptive
statistics are presented as mean (X) and standard deviation (SD).

Table S5. Genus-level statistical analysis of gut microbiota composition across experimental groups and time points (T1 and T2).

	Lactobacillus_T1_17.10.2024	Lactobacillus_T2_21.10.2024	Test Statistics
Control	35.3±0.11^{a} (35.12 - 35.42)	21.23±0.96^{a} (19.81 - 22.34)	t=32.747 p<0.001 pη2=13.369
İfosfamide	3.81±0.14^{d} (3.60 – 4.00)	17.34±0.35^{b} (16.72 - 17.63)	t=90.807 p<0.001 pη2=37.052
Gallic Asid	9.52±0.68^{c} (8.65 - 10.29)	14.19±0.24^{c} (13.86 - 14.5)	t=13.228 p<0.001 pη2=5.404
İfosfamide+Gallic Asid	14.17±0.27^{b} (13.88 - 14.58)	5.40±0.03^{d} (5.35 - 5.45)	t=83.016 p<0.001 pη2=33.891
Test Statistics	F=8065.891 p<0.001 pη2=0.999	F=992.413 p<0.001 pη2=0.993	
Statistical Model	Group Effect: F=8970.466 p<0.001 pη2=0.999<br>Time Effect: F=62.967 p<0.001 pη2=0.759<br>Grup*Time Effect: F=1848.911 p<0.001 pη2=0.996		
	Ligilactobacillus_T1_17.10.2024	Ligilactobacillus_T2_21.10.2024	
Control	6.81±0.04^{b} (6.75 - 6.86)	8.65±0.32^{a} (8.22 - 9.10)	t=12.955 p<0.001 pη2=5.289
İfosfamide	0.55±0.04^{d} (0.48 - 0.60)	8.11±0.12^{b} (7.94 - 8.28)	t=145.220 p<0.001 pη2=59.286
Gallic Asid	12.18±0.83^{a} (11.02 - 13.09)	3.44±0.06^{c} (3.36 - 3.51)	t=24.526 p<0.001 pη2=10.013
İfosfamide+Gallic Asid	1.71±0.03^{c} (1.66 - 1.75)	0.70±0.03^{d} (0.65 - 0.73)	t=57.510 p<0.001 pη2=23.479
Test Statistics	F=977.893 p<0.001 pη2=0.993	F2911.018 p<0.001 pη2=0.998	
Statistical Model	Group Effect: F=1309.296 p<0.001 pη2=0.995<br>Time Effect: F=0.799 p=0.382 pη2=0.038<br>Grup*Time Effect: F=124.916 p<0.001 pη2=0.995		
	Segatella_T1_17.10.2024	Segatella_T2_21.10.2024	
Control	12.35±0.05^{a} (12.28 - 12.42)	5.89±0.17^{a} (5.62 - 6.12)	t=76.066 p<0.001 pη2=31.054
İfosfamide	1.61±0.08^{d} (1.50 - 1.74)	5.08±0.04^{b} (5.02 - 5.12)	t=105.924 p<0.001 pη2=43.243
Gallic Asid	5.15±0.42^{c} (4.55 - 5.65)	5.80±0.10^{a} (5.66 - 5.92)	t=3.192 p=0.024 pη2=1.303
İfosfamide+Gallic Asid	5.97±0.14^{b} (5.79 - 6.14)	1.65±0.02^{c} (1.60 - 1.67)	t=64.438 p<0.001 pη2=26.307
Test Statistics	F= 2308.260 p<0.001 pη2=0.997	F=2347.287 p<0.001 pη2=0.997	
Statistical Model	Group Effect: F=3758.444 p<0.001 pη2=0.998<br>Time Effect: F=827.399 p<0.001 pη2=0.976<br>Grup*Time Effect: F=1529.978 p<0.001 pη2=0.996		
	Romboutsia_T1_17.10.2024)	Romboutsia_T2_21.10.2024	
Control	3.94±0.03^{c} (3.89 - 3.97)	5.54±0.25^{b} (5.22 - 5.87)	t=14.521 p<0.001 pη2=5.928
İfosfamide	5.00±0.14^{b} (4.80 - 5.20)	9.35±0.12^{a} (9.18 - 9.50)	t=55.097 p<0.001 pη2=22.493
Gallic Asid	3.31±0.26^{d} (3.01 - 3.7)	4.08±0.07^{d} (3.99 - 4.18)	t=6.261 p=0.002 pη2=2.556
İfosfamide+Gallic Asid	6.44±0.11^{a} (6.29 - 6.58)	4.76±0.03^{c} (4.70 - 4.8)	t=38.285 p<0.001 pη2=15.630
Test Statistics	F=452.788 p<0.001 pη2=0.985	F=1576.149 p<0.001 pη2=0.996	
Statistical Model	Group Effect: F=1349.855 p<0.001 pη2=0.995<br>Time Effect: F=717.710 p<0.001 pη2=0.973<br>Grup*Time Effect: F=294.762 p<0.001 pη2=0.990		
	Blautia_T1_17.10.2024	Blautia_T2_21.10.2024	
Control	2.91±0.02^{b} (2.88 - 2.94)	3.20±0.14^{a} (3.01 - 3.42)	t=4.650 p=0.006 pη2=1.898
İfosfamide	0.42±0.02^{d} (0.40 - 0.45)	1.23±0.07^{c} (1.11 - 1.3)	t=21.323 p<0.001 pη2=8.705
Gallic Asid	1.54±0.16^{c} (1.34 - 1.78)	1.67±0.03^{b} (1.63 - 1.71)	t=1.684 p=0.153 pη2=0.687

İfosfamide+Gallic Asid	5.26±0.05[a] (5.19 - 5.33)	0.53±0.02[d] (0.50 - 0.55)	t=348.771 p<0.001 pη2=142.385
Test Statistics	F=3465.224 p<0.001 pη2=0.998	F=1199.907 p<0.001 pη2=0.994	
Statistical Model	Group Effect: F=2527.174 p<0.001 pη2=0.997<br>Time Effect: F=1078.633 p<0.001 pη2=0.982<br>Grup*Time Effect: F=2355.457 p<0.001 pη2=0.997		
	Enterococcus_T1_17.10.2024	Enterococcus_T2_21.10.2024	
Control	0.96±0.02[b] (0.93 - 0.98)	1.04±0.05[c] (0.97 - 1.12)	t=3.220 p=0.023 pη2=1.315
İfosfamide	0.41±0.03[c] (0.37 - 0.45)	4.22±0.05[a] (4.14 - 4.28)	t=185.909 p<0.001 pη2=75.897
Gallic Asid	1.28±0.14[a] (1.11 - 1.48)	0.28±0.01[d] (0.27 - 0.29)	t=17.367 p<0.001 pη2=7.090
İfosfamide+Gallic Asid	0.24±0.01[d] (0.22 - 0.26)	2.44±0.12[b] (2.21 - 2.54)	t=47.417 p<0.001 pη2=19.358
Test Statistics	F=273.759 p<0.001 pη2=0.976	F=3508.259 p<0.001 pη2=0.998	
Statistical Model	Group Effect: F=1053.843 p<0.001 pη2=0.994<br>Time Effect: F=3909.284 p<0.001 pη2=0.995<br>Grup*Time Effect: F=2790.991 p<0.001 pη2=0.998		
	Clostridium_T1_17.10.2024	Clostridium_T2_21.10.2024	
Control	0.40±0.01[c] (0.38 - 0.42)	0.76±0.03[b] (0.72 - 0.81)	t=22.033 p<0.001 pη2=8.995
İfosfamide	0.31±0.03[d] (0.26 - 0.34)	1.17±0.09[a] (1.05 - 1.26)	t=24.845 p<0.001 pη2=10.143
Gallic Asid	0.54±0.06[b] (0.46 - 0.62)	0.66±0.04[c] (0.60 - 0.71)	t=3.406 p=0.019 pη2=1.391
İfosfamide+Gallic Asid	1.18±0.04[a] (1.13 - 1.24)	1.17±0.06[a] (1.07 - 1.24)	t=0.369 p=0.727 pη2=0.151
Test Statistics	F=570.290 p<0.001 pη2=0.988	F=121.507 p<0.001 pη2=0.948	
Statistical Model	Group Effect: F=443.847 p<0.001 pη2=0.995<br>Time Effect: F=434.901 p<0.001 pη2=0.956<br>Grup*Time Effect: F=144.893 p<0.001 pη2=0.956		
	Escherichia_T1_17.10.2024	Escherichia_T2_21.10.2024	
Control	0.25±0.01[d] (0.23 - 0.27)	0.36±0.02[c] (0.34 - 0.39)	t= 10.752 p<0.001 pη2=4.389
İfosfamide	0.38±0.03[b] (0.34 - 0.42)	0.40±0.02[b] (0.37 - 0.43)	t=1.732 p=0.144 pη2=0.707
Gallic Asid	0.44±0.02[a] (0.40 - 0.47)	0.26±0.01[d] (0.25 - 0.26)	t=18.656 p<0.001 pη2=7.616
İfosfamide+Gallic Asid	0.28±0.02[c] (0.25 - 0.31)	0.43±0.02[a] (0.39 - 0.46)	t=27.336 p<0.001 pη2=11.160
Test Statistics	F=87.532 p<0.001 pη2=0.929	F=99.651 p<0.001 pη2=0.937	
Statistical Model	Group Effect: F=24.825 p<0.001 pη2=0.788<br>Time Effect: F=25.104 p<0.001 pη2=0.557<br>Grup*Time Effect: F=240.488 p<0.001 pη2=0.973		
	Bacteroides_T1_17.10.2024	Bacteroides_T2_21.10.2024	
Control	0.56±0.02[c] (0.53 - 0.58)	0.99±0.05[c] (0.93 - 1.06)	t=19.977 p<0.001 pη2=8.156
İfosfamide	1.11±0.07[b] (1.00 - 1.20)	0.58±0.01[d] (0.56 - 0.6)	t=18.428 p<0.001 pη2=7.523
Gallic Asid	0.62±0.08[c] (0.52 - 0.72)	1.07±0.02[b] (1.04 - 1.1)	t=11.405 p<0.001 pη2=4.656
İfosfamide+Gallic Asid	2.04±0.09[a] (1.88 - 2.12)	2.07±0.08[a] (1.95 - 2.18)	t=1.611 p=0.168 pη2=0.658
Test Statistics	F=586.523 p<0.001 pη2=0.989	F=1085.186 p<0.001 pη2=0994	
Statistical Model	Group Effect: F=987.324 p<0.001 pη2=0.993<br>Time Effect: F=47.097 p<0.001 pη2=0.702<br>Grup*Time Effect: F=265.500 p<0.001 pη2=0.976		

t: paired simple t-test, KW: Kruskal-Wallis, Z: Wilcoxon, F: Mixed Design ANOVA, pη2: Partial Eta Squared, a>b>c>d: Differences between means with different letters in the same row or column are significant ($p<0.05$). Descriptive
statistics are presented as mean (X) and standard deviation (SD).

Table S6. Species-level statistical analysis of gut microbiota composition across experimental groups and time points (T1 and T2).

	Lactobacillus johnsonii _T1_17.10.2024 *X ± SS*	*Lactobacillus johnsonii* _T2_21.10.2024 *X ± SS*	Test Statistics
Control	30.79±0.24^{a} (30.48 - 31.14)	12.52±0.79^{a} (11.62 - 13.61)	t=64.125 p<0.001 pη2=26.179
İfosfamidee	2.50±0.08^{d} (2.39 - 2.61)	11.16±0.31^{b} (10.71 - 11.55)	t=56.191 p<0.001 pη2=22.940
Gallic Asid	6.01±0.28^{b} (5.51 - 6.26)	10.59±0.03^{c} (10.54 - 10.63)	t=43.758 p<0.001 pη2=17.864
İfosfamidee+Gallic Asid	5.35±0.52^{c} (4.33 - 5.81)	4.06±0.03^{d} (4.02 - 4.10)	t=6.114 p=0.002 pη2=2.496
Test Statistics	F=10186.166 p<0.001 pη2=0.999	F=469.845 p<0.001 pη2=0.986	
Statistical Model	Group Effect: F=4309.189 p<0.001 pη2=0.998<br>Time Effect: F=248.708 p<0.001 pη2=0.926<br>Group*Time Effect: F=3505.446 p<0.001 pη2=0.998		
	Ligilactobacillus murinus T1_17.10.2024	*Ligilactobacillus murinus* T2_21.10.2024	
Control	3.21±0.03^{b} (3.19 - 3.28)	6.01±0.32^{a} (5.76 - 6.56)	Z=2.201 p=0.028 pη2=8.492
İfosfamidee	0.32±0.02^{d} (0.30 - 0.35)	5.26±0.14^{b} (5.05 - 5.44)	t=78.670 p<0.001 pη2=32.117
Gallic Asid	8.78±0.38^{a} (8.12 - 9.24)	2.10±0.10^{c} (1.98 - 2.22)	t=37.976 p<0.001 pη2=15.504
İfosfamidee+Gallic Asid	1.09±0.03^{c} (1.04 - 1.13)	0.41±0.02^{d} (0.39 - 0.43)	t=55.761 p<0.001 pη2=22.764
Test Statistics	KW=21.628 p<0.001 pη2=0.997	KW=21.609 p<0.001 pη2=0.995	
Statistical Model	Group Effect: F=1758.583 p<0.001 pη2=0.996<br>Time Effect: F=2.622 p=0.121 pη2=0.116<br>Grup*Time Effect: F=1938.308 p<0.001 pη2=0.997		
	Segatella copri T1_17.10.2024	*Segatella copri* T2_21.10.2024	
Control	9.68±0.10^{a} (9.55 - 9.81)	4.44±0.22^{a} (4.06 - 4.72)	t=54.423 p<0.001 pη2=22.218
İfosfamidee	1.15±0.03^{c} (1.11 - 1.20)	4.22±0.08^{b} (4.11 - 4.33)	t=139.163 p<0.001 pη2=56.813
Gallic Asid	4.19±0.15^{b} (3.94 - 4.38)	4.65±0.27^{a} (4.34 - 5.05)	t=3.635 p=0.015 pη2=1.484
İfosfamidee+Gallic Asid	4.29±0.12^{b} (4.10 - 4.44)	1.12±0.02^{c} (1.09 - 1.15)	t=66.583 p<0.001 pη2=27.183
Test Statistics	F=6294.214 p<0.001 pη2=0.999	F=518.093 p<0.001 pη2=0.987	
Statistical Model	Group Effect: F=2180.566 p<0.001 pη2=0.997<br>Time Effect: F=862.249 p<0.001 pη2=0.977<br>Grup*Time Effect: F=1982.877 p<0.001 pη2=0.997		
	Romboutsia ilealis _T1_17.10.2024	*Romboutsia ilealis* _T2_21.10.2024	
Control	3.63±0.03^{c} (3.60 - 3.68)	5.06±0.16^{b} (4.84 - 5.33)	Z=2.201 p=0.028 pη2=8.193
İfosfamidee	4.35±0.10^{b} (4.21 - 4.48)	8.15±0.19^{a} (7.93 - 8.48)	t=64.049 p<0.001 pη2=26.148
Gallic Asid	3.12±0.63^{d} (2.29 - 4.01)	3.40±0.01^{d} (3.38 - 3.41)	t=1.070 p=0.333 pη=0.437
İfosfamidee+Gallic Asid	5.48±0.19^{a} (5.22 - 5.77)	4.24±0.03^{c} (4.20 - 4.28)	t=18.315 p<0.001 pη2=7.447
Test Statistics	F=55.779 p<0.001 pη2=0.893	KW=21.628 p<0.001 pη2=0.996	
Statistical Model	Group Effect: F=270.564 p<0.001 pη2=0.976<br>Time Effect: F=255.756 p<0.001 pη2=0.919<br>Group*Time Effect: F=223.478 p<0.001 pη2=0.971		

	Lactobacillus İntestinalis T1_17.10.2024	*Lactobacillus İntestinalis* T2_21.10.2024	
Control	2.90±0.03^{c} (2.88 - 2.95)	7.99±0.64^{a} (7.19 - 8.7)	Z=2.201 p=0.028 pη2=8.093
İfosfamidee	1.20±0.03^{d} (1.16 - 1.25)	5.07±0.10^{b} (4.90 - 5.19)	t=110.927 p<0.001 pη2=45.286
Gallic Asid	4.06±0.16^{b} (3.80 - 4.27)	2.58±0.17^{d} (2.33 - 2.82)	t=16.859 p<0.001 pη2=6.883
İfosfamidee+Gallic Asid	7.85±0.23^{a} (7.50 - 8.11)	4.06±0.03^{c} (4.02 - 4.10)	t=35.383 p<0.001 pη2=15.450
Test Statistics	KW=21.638 p<0.001 pη2=0.997	KW=21.600 p<0.001 pη2=0.976	
Statistical Model	Grup Etkisi: F=364.404 p<0.001 pη2=0.982 Time Effect: F= 157.509 p<0.001 pη2=0.887 Grup*Time Effect: F=834.492 p<0.001 pη2=0.992		
	Escherichia coli _T1_17.10.2024	*Escherichia coli* _T2_21.10.2024	
Control	0.24±0.02^{d} (0.22 - 0.27)	0.37±0.01^{c} (0.35 - 0.39)	t= 13.969 p<0.001 pη2=5.703
İfosfamidee	0.40±0.01^{b} (0.38 - 0.42)	0.40±0.01^{b} (0.38 - 0.41)	t= 0.164 p=0.876 pη2=0.067
Gallic Asid	0.43±0.03^{a} (0.39 - 0.46)	0.25±0.02^{d} (0.23 - 0.27)	t=14.540 p<0.001 pη2=5.936
İfosfamidee+Gallic Asid	0.27±0.01^{c} (0.25 - 0.28)	0.43±0.03^{a} (0.39 - 0.47)	t= 17.234 p<0.001 pη2=7.036
Test Statistics	F=162.428 p<0.001 pη2=0.961	F=108.077 p<0.001 pη2=0.942	
Statistical Model	Group Effect: F=49.884 p<0.001 pη2=0.882 Time Effect: F=31.704 p<0.001 pη2=0.613 Grup*Time Effect: F=224.929 p<0.001 pη2=0.971		
	Enterococcus hirae _T1_17.10.2024	*Enterococcus hirae* _T2_21.10.2024	
Control	0.23±0.02a (0.21 - 0.26)	0.23±0.01^{b} (0.21 - 0.25)	t=0.307 p=0.771 pη=0.125
İfosfamidee	0.20±0.01b (0.18 - 0.21)	0.66±0.41^{a} (0.47 - 1.50)	Z=2.201 p=0.028 pη2=1.121
Gallic Asid	0.12±0.03c (0.09 - 0.17)	0.33±0.02^{b} (0.29 - 0.36)	t=34.709 p<0.001 pη2=14.170
İfosfamidee+Gallic Asid	0.09±0.00d (0.08 - 0.09)	0.42±0.01^{ab} (0.41 - 0.43)	t=98.078 p<0.001 pη2=40.040
Test Statistics	KW=21.294 p<0.001 pη2=0.928	F=4.774 p=0.011 pη2=0.417	
Statistical Model	Group Effect: F=5.261 p=0.008 pη2=0.441 Time Effect: F=35.582 p<0.001 pη2=0.640 Grup*Time Effect: F=5.474 p=0.007 pη2=0.451		

t: paired simple t-test, KW: Kruskal-Wallis, Z: Wilcoxon, F: Mixed Design ANOVA, pη2: Partial Eta Squared, a>b>c>d: Differences between means with different letters in the same row or column are significant (p<0.05). Descriptive
statistics are presented as mean (X) and standard deviation (SD).
